# Degradation of Poly(ethylene terephthalate) Catalyzed by Nonmetallic Dibasic Ionic Liquids under UV Radiation

**DOI:** 10.3390/ma17071583

**Published:** 2024-03-29

**Authors:** Ruiqi Zhang, Xu Zheng, Xiujie Cheng, Junli Xu, Yi Li, Qing Zhou, Jiayu Xin, Dongxia Yan, Xingmei Lu

**Affiliations:** 1 Beijing Key Laboratory of Ionic Liquids Clean Process, CAS Key Laboratory of Green Process and Engineering, State Key Laboratory of Multiphase Complex Systems, Institute of Process Engineering, Chinese Academy of Sciences, Beijing 100190, China; rqzhang@ipe.ac.cn (R.Z.); jyxin@ipe.ac.cn (J.X.);; 2 School of Chemical Engineering, University of Chinese Academy of Sciences, Beijing 100049, China; 3 College of Chemistry, Liaoning University, Shenyang 110036, China

**Keywords:** PET glycolysis, nonmetallic dibasic ionic liquids, UV radiation, catalyst

## Abstract

Nonmetallic ionic liquids (ILs) exhibit unique advantages in catalyzing poly (ethylene terephthalate) (PET) glycolysis, but usually require longer reaction times. We found that exposure to UV radiation can accelerate the glycolysis reaction and significantly reduce the reaction time. In this work, we synthesized five nonmetallic dibasic ILs, and their glycolysis catalytic activity was investigated. 1,8-diazabicyclo [5,4,0] undec-7-ene imidazole ([HDBU]Im) exhibited better catalytic performance. Meanwhile, UV radiation is used as a reinforcement method to improve the PET glycolysis efficiency. Under optimal conditions (5 g PET, 20 g ethylene glycol (EG), 0.25 g [HDBU]Im, 10,000 µW·cm^−2^ UV radiation reacted for 90 min at 185 °C), the PET conversion and BHET yield were 100% and 88.9%, respectively. Based on the UV-visible spectrum, it was found that UV radiation can activate the C=O in PET. Hence, the incorporation of UV radiation can considerably diminish the activation energy of the reaction, shortening the reaction time of PET degradation. Finally, a possible reaction mechanism of [HDBU]Im-catalyzed PET glycolysis under UV radiation was proposed.

## 1. Introduction

PET has an important position in the field of plastic applications since it is non-toxic, odorless, and tasteless [1]. These unique qualities allow PET to be employed in diverse textiles and soft drink bottles [2,3]. However, it poses a serious threat to both the environment and ecosystems due to the accumulation of large quantities of PET waste [4]. Furthermore, while PET itself is considered nontoxic, certain additives and colorants present in PET products have the potential to accumulate in the body and lead to toxicity in organisms [5]. And under 100% humidity conditions, the life expectancy of a PET bottle is approximately 93 years [6]. Therefore, achieving complete degradation of PET in natural environments is an almost impossible challenge. In addition, approximately seven barrels of petroleum resources can be saved for every ton of mixed plastic recycled [7]. Hence, the recycling of waste PET holds significant importance for the environment, resources, and economy.

Chemical recycling of waste PET has garnered increasing attention as a means to achieve a closed-loop process and diminish the reliance on fossil energy resources. It facilitates the efficient utilization of waste PET by depolymerizing it into monomers or other chemical compounds [8,9]. Various chemical manners have been studied including glycolysis [10], hydrolysis [11], methanolysis [12], and aminolysis [13]. Among them, glycolysis has attracted great attention because of its mild reaction conditions and because no toxic substances are released during the reaction. PET glycolysis is a process of transesterification with EG at a certain temperature to generate bis (hydroxyethyl) terephthalate (BHET). In the absence of a catalyst, the reaction takes place very slowly [14]. Therefore, various glycolysis catalysts have been developed [15]. Some catalysts, such as ZnO [16], Mn_3_O_4_ [16], zeolite [17], and SO_4_^2−^/ZnO [18] have been adopted, but they can not dissolve in EG [19]. Thus, the catalytic activity is relatively low due to the complexity of mass transfer. Compared with these solid catalysts, ILs exhibit superior catalytic activity. In 2009, our team first used [Bmim]Cl as a solvent to dissolve and degrade PET [20]. Since then, more and more imidazolium-based ILs have been adopted, such as [Bmim]_2_[CoCl_4_] [21], [Amim][ZnCl_3_] [22], and [Bmim][ZnCl_3_] [23]. Similar to metal salts, ILs that contain Zn^2+^ exhibit excellent catalytic efficiency. However, the residual Zn^2+^ in BHET can seriously downgrade the quality of BHET and recycled PET. Therefore, to solve this issue, some nonmetallic ILs catalysts were designed, such as [Ch][OAc] [24] and [Ch]_3_[PO_4_] [25]. All of them are biocompatible ILs, but it takes more than three hours to fully degrade PET, which leads to high energy consumption and is not conducive to large-scale production. Therefore, it is imperative to improve the reaction rate of nonmetallic IL catalysts. Previous researchers have indicated that alkaline ILs often exhibit better catalytic activity than acidic ILs in PET glycolysis reactions. The catalytic performance of DBU, which has stronger alkalinity, has been demonstrated [26]. Additionally, imidazole anion ([Im]-) also exhibits alkalinity. Therefore, it is considered desirable to design and synthesize [HDBU]Im as a nonmetallic dibasic catalyst, with the anticipation of showcasing remarkable catalytic efficacy in the PET glycolysis reaction.

On the other hand, researchers have developed various auxiliary promotion methods to further enhance PET glycolysis reactions. Alnaqbi et al. [27] used [Bmim]Br as a catalyst for PET glycolysis under microwaves, which could reduce the glycolysis time from nearly 9 h (conventional heating) to 2 h, dramatically increasing the catalytic reaction rate. Imran et al. [28] utilized supercritical ethylene glycol (450 °C and 15.3 MPa) for PET degradation, resulting in a BHET yield of 93.5% within 30 min. The shortened reaction time is attributed to its solubility and high solvent density. However, the temperature and pressure under supercritical conditions are relatively high. Le et al. [29] developed a co-solvent-assisted PET glycolysis method that can complete PET decomposition by reacting at 153 °C for 2 h using anisole as a co-solvent. However, anisole has a detrimental impact on the environment. As part of the sunlight spectrum, ultraviolet (UV) radiation is cheaper, more accessible, and more environmentally friendly than other auxiliary promotion methods. UV radiation has been identified as the primary factor for plastic degradation in the natural environment [30]. MacLeod et al. [31] reported that under marine conditions, the photo-induced oxidation of PET is likely to occur, which leads to a reduction in molecular weight. In this process, the function of UV radiation is to induce the cleavage of the carbon–carbon backbone, leading to chain scission. Lee et al. [32] identified the photo-degradation products of PET films including esters, peresters, and benzoic acids. More et al. [33] applied UV radiation in PET aminolysis. It can be found that the speed of the degradation process was enhanced, which was due to the UV radiation attacking the ester linkage of the PET [33]. Therefore, UV radiation can be used as an efficacious method to promote the occurrence of PET degradation. However, the research on IL-catalyzed PET glycolysis under UV radiation-assisted systems is still limited. Motivated by this, UV radiation was introduced into the PET degradation system to enhance the catalytic reaction rate of nonmetallic ILs and the yield of BHET.

In this study, UV radiation was introduced into the IL-catalyzed PET glycolysis system as an auxiliary strengthening method. Meanwhile, five nonmetallic dibasic ILs catalysts were synthesized, and their activity was tested under the same conditions. Among them, [HDBU]Im exhibited the best activity. The optimization of the reaction conditions was achieved through the evaluation of influencing parameters. The reaction kinetics under UV radiation and without UV radiation were studied, and the impact of UV radiation on the reaction mechanism was subsequently examined. Finally, based on the experimental findings a potential degradation mechanism was suggested. Compared with conventional heating methods, UV radiation can shorten the reaction time of nonmetallic IL-catalyzed PET glycolysis and increase the yield of BHET. This approach conserves energy and processing expenses without using hazardous reagents, offering a novel strategy for enhancing the efficiency and sustainability of PET chemical recycling.

## 2. Materials and Methods

### 2.1. Materials

Raw PET material was acquired from DuPont (Wilmington, DE, USA). They were smashed to 40–60 mesh. At this particle size, PET has a large surface area, which is conducive to mutual contact between PET, EG, and IL, which can speed up the reaction rate and achieve faster degradation of PET. The molecular weight (M_n_) of the PET powder was 4.35 × 10^4^ g·mol^−1^, determined using GPC analysis. Imidazole (Im), 2-methylimidazole (2-MeIm), 2-ethylimidazole (2-EtIm), 4-methylimidazole (4-MeIm), and 2-ethyl-4-methylimidazole (2-Et-4-MeIm) were purchased from Sinopharm Chemical Reagent Beijing Co., Ltd., Beijing, China.

### 2.2. Synthesis of ILs

A series of nonmetallic dibasic ILs were synthesized according to reported procedures [34,35]. Taking [HDBU]Im as an example, it was synthesized through the neutralization reaction of DBU and imidazole. A certain amount of imidazole was added dropwise to the anhydrous ethanol solution of DBU. The molar amount of imidazole in the mixture is equimolar to DBU. DBU, imidazole, and anhydrous ethanol were stirred vigorously at 25 °C for 24 h. The anhydrous ethanol was removed using vacuum evaporation, and a pale yellow transparent liquid was obtained. The synthesis steps of 1,8-diazabicyclo [5,4,0] undec-7-ene 2-methylimidazole ([HDBU][2-MeIm]), 1,8-diazabicyclo [5,4,0] undec-7-ene 2-ethylimidazole ([HDBU][2-EtIm]), 1,8-diazabicyclo [5,4,0] undec-7-ene 4-methylimidazole ([HDBU][4-MeIm]), and 1,8-diazabicyclo [5,4,0] undec-7-ene 4-methylimidazole 2-ethyl-4-methylimidazole ([HDBU][2-Et-4-MeIm]) were similar to [HDBU]Im.

### 2.3. PET Glycolysis under UV Radiation

The reaction was conducted in a dark box under UV radiation. In each experiment, a 50 mL three-neck flask containing a thermometer, a condensation device, and a magnetic stirrer was utilized, and 5.0 g PET powder, 20 g EG, and a definite amount of catalyst were added. They were heated in an oil bath. The temperature was from 160 °C to 195 °C and the reaction time was from 20 min to 150 min. After the completion of degradation, the mixture was cooled to 25 °C, then poured into 500 mL deionized water and stirred vigorously at 70 °C for 1 h to separate the unreacted PET particles through filtration. The unreacted PET was dried at 65 °C for 12 h, and it was weighted to compute PET conversion, which was defined using Equation (1)

(1) $$\mathrm{PET}\;\mathrm{Conversion}=\frac{W_{0}\;-\;W_{1}}{W_{0}}\times 100\%$$

where W_0_ represents the initial weight of the PET and W_1_ represents the weight of the unreacted PET. The filtrate was subsequently subjected to rotary evaporation in a vacuum at 65 °C, and the concentrated filtrate was cooled at 4 °C for 12 h. Finally, a needle-like BHET monomer was obtained through filtration and drying. The BHET yield is defined using Equation (2):

(2) $$\mathrm{BHET}\;\mathrm{Yield}=\frac{W_{\mathrm{BHET}}/M_{\mathrm{BHET}}}{W_{0}/M_{\mathrm{PET}}}$$

where W_BHET_ is the weight of BHET and M_BHET_ and M_PET_ are the molecular weight of BHET and the PET repeat unit, respectively. 

### 2.4. Recycling of the EG and Catalyst

After the BHET was extracted through filtration, the remaining EG and catalyst in the filtrate were subjected to rotary evaporation at 65 °C and then stored in a vacuum oven at 70 °C for over 12 h to remove as much water as possible. In the succeeding cycle, a certain amount of the EG was added to ensure that the quantity of the solution was equal to 20 g.

### 2.5. Characterization

The ^1^H NMR spectra were analyzed using an AVANCE-III 600 NMR spectrometer (Bruker, Fällanden, Switzerland), operated at 600 MHz in CDCl_3_ and DMSO-d_6_. The FT-IR testing of the catalysts and main product were performed with a Nicolet 380 FT-IR spectrograph (Thermo Nicolet, Waltham, MA, USA). The differentials scanning calorimeter (DSC) STARe system was applied to generate the DSC curve from 25 °C to 200 °C at the heating rate of 10 °C/min under a nitrogen atmosphere. The thermogravimetric analysis (TGA) curve was tested using DTG-60H (Shimadzu, Tokyo, Japan) under the nitrogen atmosphere, and the samples were heated from 25 °C to 600 °C at the heating rate of 10 °C/min. Gel permeation chromatography (GPC) (Agilent, PL-50, Santa Clara, CA, USA) was applied to determine the molecular weights of PET pellets and oligomers. The testing condition was as follows: the oven temperature was 30 °C, the mobile phase was chloroform.

## 3. Results

### 3.1. Screening of Catalysts

**Scheme 1:**
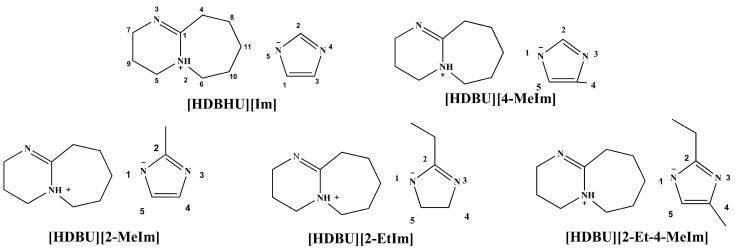
Molecular formula of DBU-based ionic liquids (atoms were numbered).

To investigate the effect of imidazole derivatives anions on catalytic activity, a series of nonmetallic dibasic ILs were prepared. Their structures are shown in Scheme 1. The steric-hindrance effect of anions can significantly affect the catalytic activity. From Table 1, it is evident that [HDBU][Im] exhibits the highest catalytic activity, with a BHET yield of 82.9%. It was also found that when the position of 2-H, 4-H, and 5-H of the imidazole ring are replaced by other functional groups, the catalytic activity can be reduced. For entry 2 and entry 3, the catalytic activity is similar. Therefore, the position of the substituent group on the imidazole ring has relatively tiny influence on catalytic activity. For entry 3 and entry 4, if the volume of the substituent group is bigger, the steric-hindrance effect is noticeable and the hydrogen bonds between the catalyst and the EG will be weakened. Meanwhile, the electron density will be reduced. Thus, the catalytic activity decreases. It can be proved that the 2-H of the imidazole ring plays a crucial role in catalyzing PET glycolysis. 2-H is more active than others, which can leave the imidazole ring and become H^+^. The H^+^ can attack the carbon of C=O in PET, and then the electrophilicity will be improved. Thus, [HDBU][Im] is the best catalyst, and the structure of the [HDBU][Im] was characterized using FT-IR and ^1^H NMR (see Supplementary Materials). On the other hand, through a series of characterizations of the main product, it was proved that the main product obtained from the degradation of PET catalyzed by [HDBU][Im] is the monomer BHET (see Supplementary Materials for more detail).

### 3.2. The Effect of UV Radiation

**Figure 1:**
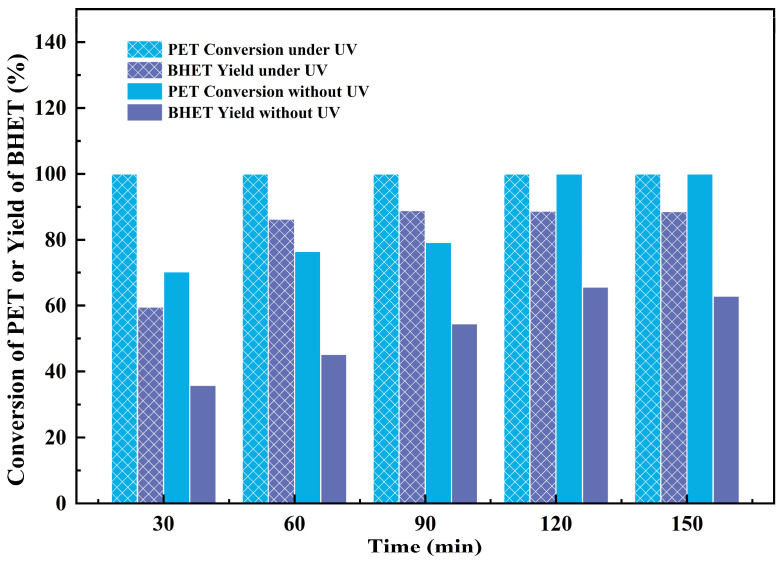
Effect of UV on PET conversion and BHET yield at different reaction time (reaction conditions: 5 g PET, 5 wt% catalyst/PET ratio, 1/4 PET/EG weight ratio at 185 °C).

In order to explore the effect of UV radiation on this reaction, the conversion of PET and the yield of BHET were compared with and without UV radiation. The experimental data are shown in Figure 1. UV radiation has been shown to enhance the depolymerization reaction of PET, resulting in a 100% conversion rate after 30 min compared to 120 min without UV radiation. Therefore, UV radiation can significantly accelerate the reaction and shorten the reaction time, and it can also increase the yield of BHET. This may be because UV radiation can degrade the polymer into oligomers effectively. To verify this conjecture, the oligomers generated under UV radiation and without UV were separated and analyzed using GPC (Table 2). UV radiation reduced the M_n_ of oligomers by approximately 50%. This shows that UV does accelerate polymer degradation, so more oligomers with lower molecular weight were produced. Son et al. [36] found that UV radiation can reduce the activation energy of the reaction system. Therefore, it is speculated that in this glycolysis reaction, UV radiation can also reduce the activation energy, accelerating the reaction rate. The effect of UV radiation on activation energy will be discussed in detail in subsequent sections.

### 3.3. Influence of Reaction Conditions

**Figure 2:**
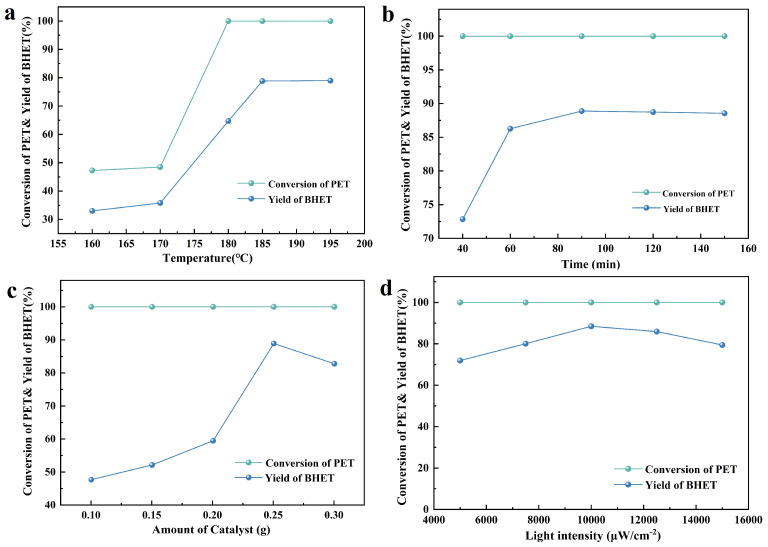
Effect of temperature (**a**) (reaction conditions: 5 g PET, 5 wt% catalyst/PET ratio, 1/4 PET/EG weight ratio with 5000 µW·cm^−2^ UV radiation for 40 min), time (**b**) (reaction conditions: 5 g PET, 5 wt% catalyst/PET ratio, 1/4 PET/EG weight ratio with 5000 µW·cm^−2^ UV radiation at 185 °C), catalyst dosage (**c**) (reaction conditions: 5 g PET, 1/4 PET/EG weight ratio with 5000 µW·cm^−2^ UV radiation at 185 °C for 90 min), and UV radiation intensity (**d**) (reaction conditions: 5 g PET, 5 wt% catalyst/PET ratio, 1/4 PET/EG weight ratio at 185 °C for 90 min) on PET degradation.

To determine the optimal reaction conditions for [HDBU][Im], various factors including temperature, reaction time, catalyst amount, and UV radiation intensity were explored. The effect of the reaction temperature is shown in Figure 2a. Below 150 °C, BHET is barely detectable. As the temperature increases, the conversion rate of PET and yield of BHET increase sharply. From 180 °C to 195 °C, the conversion rate was maintained at 100% and the BHET yield remained steady up to 78.96% from 185 °C to 195 °C. It was obvious that the transesterification reaction is an endothermic reaction, and the reaction temperature played a crucial role in the degradation of PET, so the increase in temperature was conducive to the progress of the reaction. The BHET yields at 185 °C and 195 °C were 78.82% and 78.96%, respectively. At these two temperatures, there is no significant difference in the yield of BHET. It is worth noting that as the temperature rises, so does the energy consumption. Therefore, in terms of energy conservation, 185 °C is the optimal temperature.

The effect of the reaction time is shown in Figure 2b. It is observed that from 40 min to 150 min, the conversion of PET was maintained at 100%. Under UV radiation, the PET conversion reached 100% within 40 min. Degradation was quicker than in conventional experimental conditions [21]. In this process, the conversion of PET reached 100%, while the yield of BHET demonstrated a notable increase as the reaction time was prolonged from 40 min to 90 min. Therefore, we speculated that PET was first degraded into oligomers and then the oligomers turned into BHET. Furthermore, the glycolysis of PET is a reversible reaction, with a dynamic equilibrium existing between the BHET and the oligomers. The reaction reached equilibrium after 90 min; further extending the reaction time did not enhance the yield of BHET. Thus, 90 min was determined as optimal. 

Figure 2c illustrates the effect of the amount of catalyst. PET is difficult to degrade without a catalyst. As the [HDBU]Im increased, the PET conversion remained at 100%. However, as the [HDBU]Im dosage increased from 0.1 g to 0.25 g, the yield of BHET increased significantly. The BHET yield attained its maximum value when the catalyst dosage reached 0.25 g, and the BHET yield decreased marginally with further increase of catalyst dosage. This may be due to the excessive amount of catalyst boosting the reverse reaction, causing more BHET to be converted into oligomers. Therefore, excessive catalyst dosage is not conducive to the acquisition of BHET; the optimized catalyst amount is 0.25 g. 

Figure 2d illustrates the effect of UV radiation intensity. As the UV radiation intensity increased from 5000 µW·cm^−2^ to 15,000 µW·cm^−2^, the PET conversion remained at 100%. However, UV radiation intensity can markedly affect BHET yield. When the intensity was raised from 5000 µW·cm^−2^ to 10,000 µW·cm^−2^, the BHET yield increased gradually. Based on the experimental results above, it can be deduced that UV radiation has the capability to facilitate the breakage of PET molecular chains, thereby accelerating the decomposition of PET into oligomers and monomers. After that, as the intensity was further increased, the yield of BHET decreased. It has been previously suggested that photo-grafting polymerization can occur under UV radiation for compounds containing carbonyl or ester groups [37]. When the intensity of UV radiation exceeds 10,000 µW·cm^−2^, it has the potential to polymerize BHET into oligomers [38], leading to a decrease in the yield of BHET. Therefore, 10,000 µW·cm^−2^ is the optimal UV radiation intensity, with a BHET yield of 88.9%.

### 3.4. Recycling of Solvent and Catalyst

**Figure 3:**
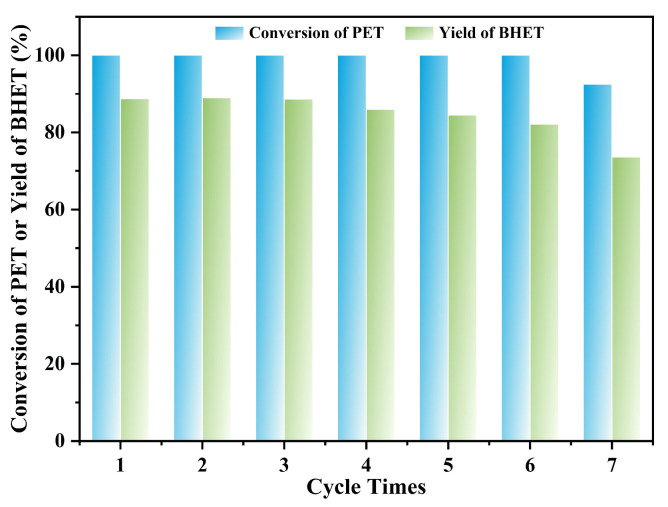
Recycling of [HDBU][Im].

Recycling of the remaining EG and catalyst is an important issue for environmental and economic reasons. The recycling ability of the catalyst is also crucial for industrialization. It was tested under optimal experimental conditions (5 g PET, 0.25 g IL, 20 g EG, 90 min, 185 °C, 10,000 µW·cm^−2^). The recycling results are shown in Figure 3, which demonstrates that [HDBU][Im] could sustain its catalytic activity when reused up to six times. After recycling seven times, the catalytic performance decreased, which may be due to the loss of [HDBU][Im] catalyst. As shown in Figure S8, the TGA curve shows that the thermal decomposition temperature of [HDBU][Im] is 201 °C. The reaction temperature of [HDBU][Im]-catalyzed PET degradation is below 201 °C, but prolonged exposure to this temperature may result in partial decomposition of the [HDBU][Im]. Therefore, the catalytic activity of [HDBU][Im] decreased after it was recycled seven times.

### 3.5. Kinetics of PET Glycolysis

Kinetics can reveal reaction processes [39]. Based on PET conversion, the kinetic of PET is first order [40,41,42]. We used the shrinking core model to calculate the activation energy of the [HDBU]Im-catalyzed PET glycolysis reaction system. To better understand the effect of UV radiation, kinetics studies with and without UV radiation were all investigated.

**Figure 4:**
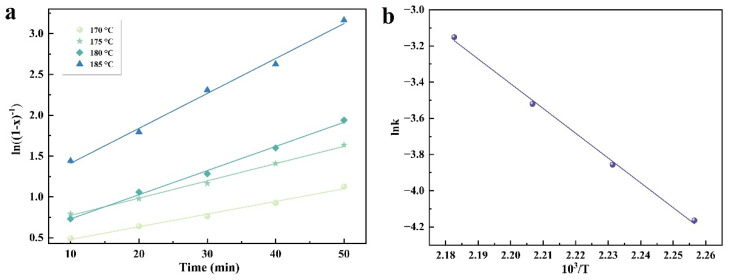
Effect of the temperature on the rate of glycolysis of PET (under UV radiation) (**a**). Arrhenius plot of the rate constant of glycolysis of PET (under UV radiation) (**b**).

Figure 4a demonstrates the effect of temperature on k under UV radiation. A rapid increase in the reaction rate constant is observed as the temperature increases from 170 °C to 185 °C, proving that temperature is an important factor. Calculated using the Arrhenius equation, the relationship between lnk and temperature is shown in Figure 4b. A fine straight line was obtained with correlation coefficient squares larger than 0.98. It indicated that PET glycolysis conforms to a first-order kinetic model. Thus, the activation energy (Ea) is 113.76 kJ·mol^−1^. 

**Figure 5:**
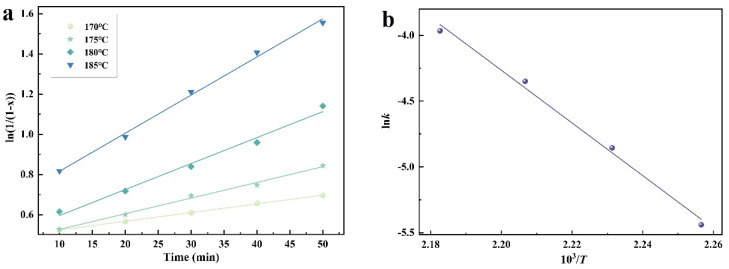
Effect of the temperature on the rate of glycolysis of PET (without UV radiation) (**a**). Arrhenius plot of the rate constant of glycolysis of PET (without UV radiation) (**b**).

Figure 5a displays the influence of temperature on k without UV radiation, and Figure 5b expresses the relationship between lnk and temperature based on the Arrhenius equation. A fine straight line was obtained with correlation coefficient squares larger than 0.98. The Ea is 166.50 kJ·mol^−1^. It is evident that UV radiation can reduce the Ea from 166.50 kJ·mol^−1^ to 113.82 kJ·mol^−1^. The Ea is defined as the minimum amount of energy necessary to initiate a chemical reaction [43]. The lower value of the Ea means the more viable a reaction is and the faster the reaction process. UV radiation can significantly reduce the activation energy of [HDBU][Im]-catalyzed PET glycolysis. Therefore, 100% conversion of PET can be achieved in a shorter time, and the yield of BHET can also be increased. 

### 3.6. PET Degradation Mechanism

To explore the catalytic mechanism of [HDBU]Im, a series of experiments were conducted under optimal conditions. The results are shown in Table 3. Obviously, the catalytic activity of [HDBU]Im is higher than imidazole or DBU. It can be inferred that [HDBU]^+^ and [Im]^−^ play a synergistic role. 

**Figure 6:**
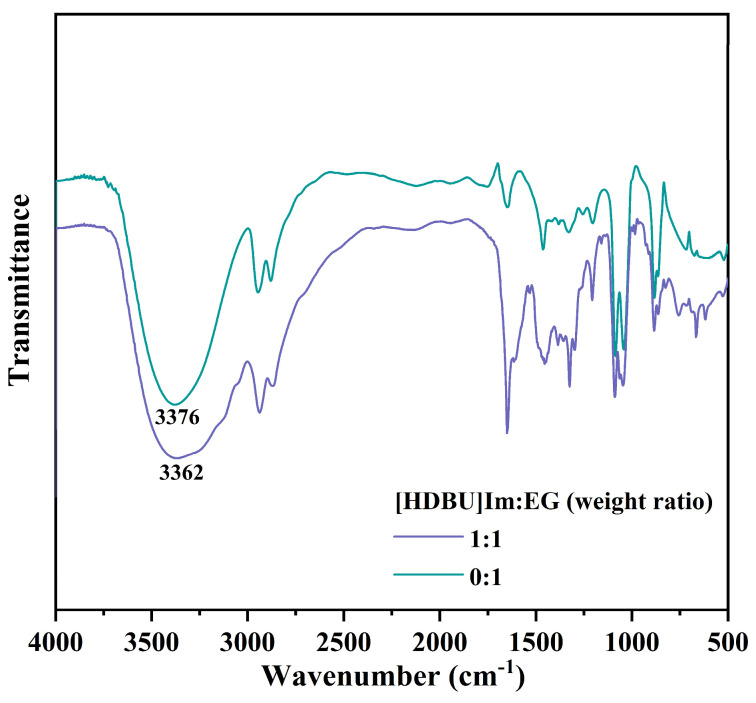
FT-IR spectra of [HDBU]Im/EG mixtures.

According to the literature, some catalysts, such as [Ch][OAc], [dimim][FeCl_4_], [Ch][Gly], and [Ch]_3_[PO_4_], can form hydrogen bonds (H-bonds) with the hydroxyl hydrogen of EG [24,25,44,45]. The H-bonds elongate the O-H bond of EG hydroxyl and enhance the electronegativity of EG oxygen [46]. Hence, EG can easily attack the carbon of the ester group in PET [21]. To reveal the interaction between [HDBU]Im and EG, FT-IR characterization was conducted for [HDBU]Im/EG mixtures (Figure 6). The O-H vibration of EG showed an obvious 14 cm^−1^ red shift as the [HDBU]Im was introduced into EG. This indicates that [HDBU]Im and EG can form H-bonds, which can effectively activate the hydroxyl group of EG. Therefore, the ability of EG to attack the ester groups in PET is enhanced. To further realize the interaction between EG and catalysts, DBU/EG mixtures were also analyzed (Figure S9). The weight ratios of DBU/EG mixtures are consistent with [HDBU]Im/EG. For DBU/EG mixtures, the red shift of EG hydroxyl vibration was 8 cm^−1^. This result further indicates that the hydrogen bonding between [HDBU][Im] and EG is stronger. Therefore, [HDBU]Im could efficiently catalyze the degradation of PET.

**Figure 7:**
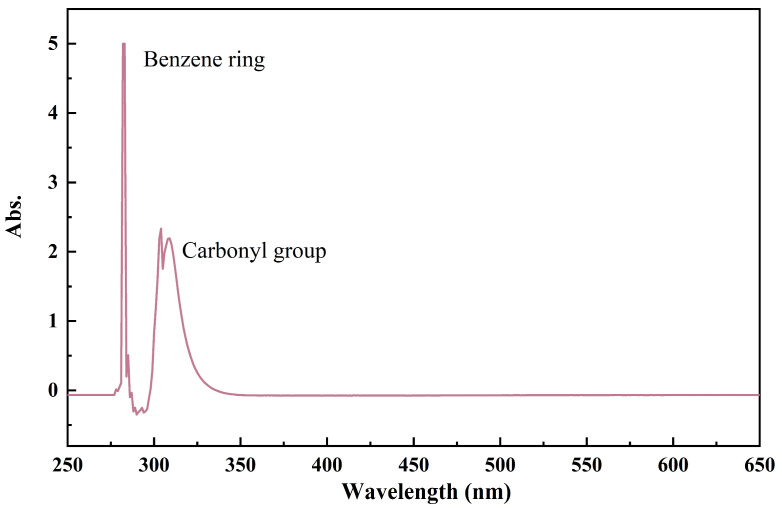
The UV-Visible Spectrum of PET.

To verify the interaction between UV radiation and PET, it is essential to study the UV absorption properties of PET (Figure 7). One strong absorption band appears at nearly 280 nm, corresponding to the benzene ring in PET [47]. The other absorption band appears at nearly 310 nm and corresponds to the carbonyl group in PET [48]. The UV absorption of the carbonyl group causes n→π*electronic transitions. The transition responsible for UV absorption in the carbonyl group can be traced to the lone pair of electrons on the O atom. One of the electrons in a lone pair can be excited to an empty π* orbital of the carbonyl group [49], so the electronegativity of the oxygen of C=O in PET becomes higher. Meanwhile, the electrophilicity of the carbon of the C=O group in PET is enhanced. It makes the carbonyl group more vulnerable to EG attacks. As a result, the effect of UV radiation is to activate the carbonyl group, which can promote PET glycolysis. The activation effect is verified by the experimental results in Section 3.5.

**Scheme 2:**
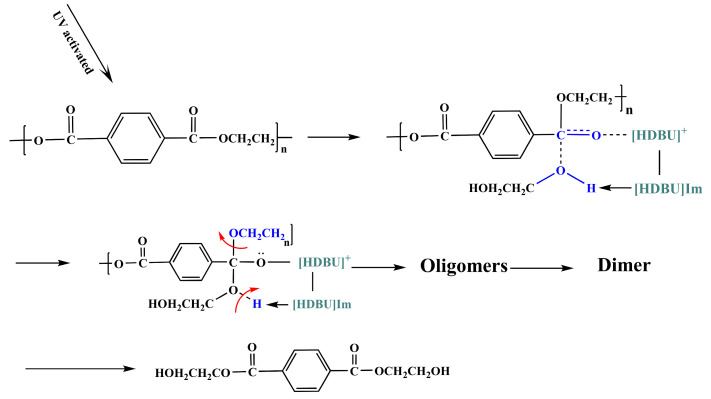
Mechanism of the glycolysis of PET catalyzed by [HDBU]Im.

In addition, the crystallinity of PET during the reaction process was investigated (Table S1), and it was found that as the degradation reaction progressed, the crystallinity of the residual PET gradually increased. This may be due to the loose arrangement of PET molecular chains in the amorphous region, which is more prone to degradation. The experimental results provide the basis for proposing the potential mechanism of PET glycolysis catalyzed by [HDBU]Im, as illustrated in Scheme 2. EG is activated by [HDBU]Im via H-bonds. The formed H-bonds make the O-H bond of EG hydroxyl longer and the electronegativity of EG oxygen higher. These cause hydrogen to be lost more easily and enhance the nucleophilicity of oxygen. Consequently, it facilitates the attack on the carbon of the PET ester group. DBU has shown relatively high catalytic activity [27]. When DBU is used as catalyst alone, it can activate the carbonyl group of the ester in PET [27]. Hence, [HDBU]^+^ can activate PET by protonating the carbon of the carbonyl group. Meanwhile, the carbonyl group in PET is also activated by UV radiation. Therefore, PET is more electrophilic, and the highly nucleophilic EG oxygen attacks the carbon of the PET ester group more easily. As the oxygen in the EG hydroxyl attacks the carbon of the ester group in PET, a tetrahedral intermediate will be formed. Thereafter, the hydrogen in EG dissipates. The acyl–oxygen bonds cleave, resulting in the detachment of the –OCH_2_CH_2_– group, which subsequently combines with H^+^ to form HOCH_2_CH_2_–. These processes are repeated to form oligomers and BHET monomers. Finally, chemical equilibrium is formed between the oligomer and the BHET.

## 4. Conclusions

In summary, we synthesized a series of nonmetallic dibasic ILs, among which [HDBU]Im showed the best performance in catalyzing PET glycolysis. The impact of UV radiation on the PET degradation was further investigated. It was discovered that the utilization of UV radiation expedited the reaction time and enhanced the yield of the monomer BHET. Afterward, the effects of different reaction conditions on PET degradation were investigated; the optimized experimental conditions were 5 g PET, 20 g EG, 0.25 g [HDBU]Im, 185 °C, 10,000 µW·cm^−2^ UV radiation reacted for 90 min, and the PET conversion and BHET yield were 100% and 88.9%, respectively. Based on reaction kinetics, UV radiation can reduce the Ea from 166.50 kJ·mol^−1^ to 113.76 kJ·mol^−1^_,_ so the rate of PET glycolysis is considerably enhanced. Throughout the reaction, UV radiation can improve the electronegativity of oxygen in C=O, so the electrophilicity of carbon in carbonyl can be enhanced. This makes PET more vulnerable to EG attacks, accelerating the catalytic reaction rate. 

This work verifies the high catalytic performance of dibasic nonmetallic ILs catalyst and UV radiation. In follow-up research, UV radiation can be considered for other reaction systems of PET degradation, aiming to reduce the reaction temperature further, achieve efficient degradation of PET under mild conditions, and provide guidance for the industrialization of PET recycling. 

## Figures and Tables

**Table 1:** Different catalysts activities for PET glycolysis ^1^.

Entry	ILs	PET Conversion (%)	BHET Yield (%)
1	[HDBU][Im]	100	82.9
2	[HDBU][4-MeIm]	100	78.3
3	[HDBU][2-MeIm]	100	77.2
4	[HDBU][2-EtIm]	96.2	74.1
5	[HDBU][2-Et-4-MeIm]	100	70.5

^1^ Reaction conditions: 5.0 g PET, 5.0 wt% catalyst, 20.0 g EG reacted for 90 min at 190 °C under 5000 µW·cm^−2^ UV radiation.

**Table 2:** GPC analysis for oligomers.

Condition	M_n_ (g∙mol^−1^)	M_w_ (g∙mol^−1^)	PD
Without UV radiation	2419	2461	1.02
With UV radiation	1084	1102	1.02

M_n_: number-average Molecular Weight, M_w_: weight-average Molecular Weight, PD = M_w_/M_n_.

**Table 3:** Catalytic activity comparison among imidazole, DBU, and [HDBU]Im ^1^.

Catalyst	Conversion of PET (%)	Yield of BHET (%)
Imidazole	22.5	14.5
DBU	100	71.5
[HDBU]Im	100	88.9

^1^ Reaction conditions: 5.0 g PET, 0.25 g catalyst, 20.0 g EG reacted for 90 min at 185 °C under 10,000 µW·cm^−2^ UV radiation.

## Data Availability

Data are contained within the article and Supplementary Materials.

## Supplementary Materials

The following supporting information can be downloaded at: https://www.mdpi.com/article/10.3390/ma17071583/s1, Figure S1: FT-IR spectrum of [HDBU][Im] [35,50,51]; Figure S2: ^1^H NMR spectrum of [HDBU][Im] [34,52]; Figure S3: ^1^H NMR spectrum of BHET [16]; Figure S4: HPLC spectrum of BHET; Figure S5: TGA curves of PET raw material and BHET [16]; Figure S6: DSC curves of BHET [21]; Figure S7: FTIR spectrum of BHET [22,40,53]; Figure S8: The TGA curve of [HDBU][Im]; Figure S9: FT-IR spectra of DBU/EG mixtures; Table S1: The changes in PET crystallinity during the degradation reaction [54,55,56,57,58].
