# Supplementary material for: Degradation of Poly(ethylene terephthalate) Catalyzed by Nonmetallic Dibasic Ionic Liquids under UV Radiation
Source: Materials (Basel). 2024 Mar 29;17(7):1583. doi: 10.3390/ma17071583 (PMC11012343; doi:10.3390/ma17071583)
Supplement: Supplementary file 1 [file materials-17-01583-s001.zip › materials-2907064-supplementary.pdf]

Supporting Information

Degradation of poly(ethylene terephthalate) catalyzed by nonmetallic dibasic ionic liquids under UV radiation

Ruiqi Zhang [1,2], Xu Zheng [3], Xiujie Cheng [1,2], Junli Xu [1,2], Yi Li [1,2], Qing Zhou [1,2], Jiayu Xin [1,2], Dongxia Yan [1,2] and Xingmei Lu [1,2,*]

1 Beijing Key Laboratory of Ionic Liquids Clean Process, CAS Key Laboratory of Green Process and Engineering, State Key Laboratory of Multiphase Complex Systems, Institute of Process Engineering, Chinese Academy of Sciences, Beijing 100190, PR China

2 College of Chemistry and Chemical Engineering, University of Chinese Academy of Sciences, Beijing 100049, PR China 3 School of Chemical Engineering, University of Chinese Academy of Sciences, Beijing 100049, P. R. China

3 College of Chemistry, Liaoning University, Shenyang 110036, P. R. China

** Correspondence: Xingmei Lu (xmlu@ipe.ac.cn)*

Section A: Characterization of the [HDBU][Im]

FT-IR and ^{1}H NMR were used to determine the structure of [HDBU][Im]. FT-IR spectra of [HDBU][Im] is shown in Fig. S1.

The peak at 3106 cm^{-1} is attributed to the stretching vibration of unsaturated C-H [1]. The peak at 2853 cm^{-1} is attributed to the stretching vibration of saturated C-H [1]. The peak at 1643 cm^{-1} is attributed to C=C stretching vibration of imidazole ring [2]. The peak at 1613 cm^{-1} is attributed to C=N stretching vibration of imidazole ring and DBU [3]. The peaks at 1353 cm^{-1} and 1368 cm^{-1} are typical stretching vibration of C=N. These above FI-IR results demonstrated the successful synthesis of [HDBU][Im].

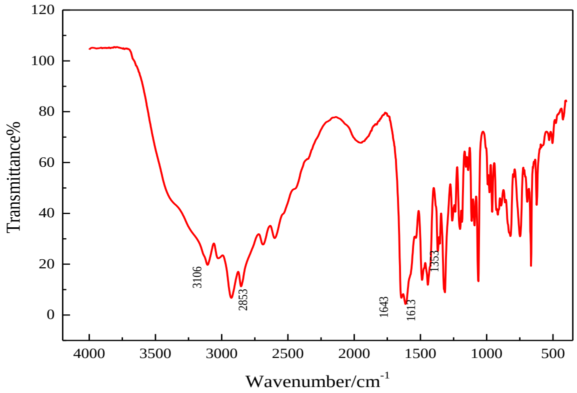

Figure S1 FT-IR spectrum of [HDBU][Im]

In the ^{1}H NMR spectrum (Fig.S2), the single peak at δ7.63 ppm corresponds to the H-14 in imidazole ring. The single peak at δ7.05 ppm represents H-12 and H-16. The triplet at δ3.26 ppm indicates H-4. The multiple peaks from δ3.18 ppm to δ3.21 ppm represent H-6 and H-7, their chemical environment is similar, so their signals cannot be separated. The single peak at δ2.38 ppm represents H-11. The multiple peaks at δ1.79 ppm corresponds to H-5. The multiple peaks at δ1.63 ppm corresponds to H-9 and H-10. The single peak at

δ1.56 ppm represents H-8. The NMR result is consistent with the data from literature, so [HDBU][Im] is successfully prepared[4,5].

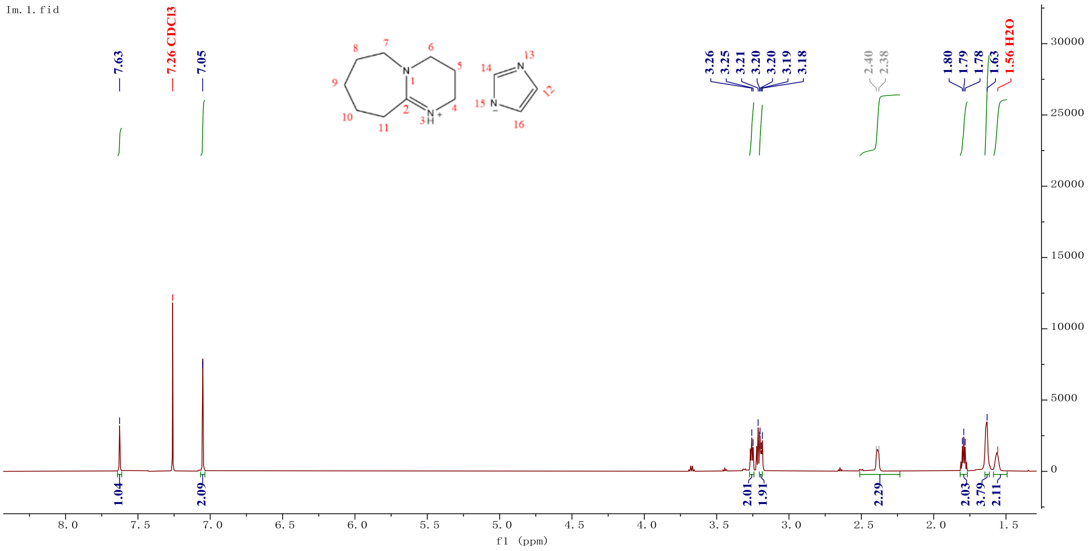

Figure S2 ^{1}H NMR spectrum of [HDBU][Im]

Section B: Characterization of the main product Thank you very much for your encouraging comments.

The structure of the main product obtained by glycolysis was determined by NMR, HPLC, TGA, DSC and FTIR. ^{1}H NMR spectrum was shown in Fig. S3. The signal at δ8.13 ppm indicates the presence of the four aromatic protons of the benzene ring (H-1). The signal at δ4.32 ppm δ3.73 ppm represents the methylene protons of COO-CH_2 (H-2) and CH_2-OH (H-3), respectively. The signal at δ4.97 ppm is the characteristic of the protons of the hydroxyl (H-4). It was demonstrated that the main product was BHET[6].

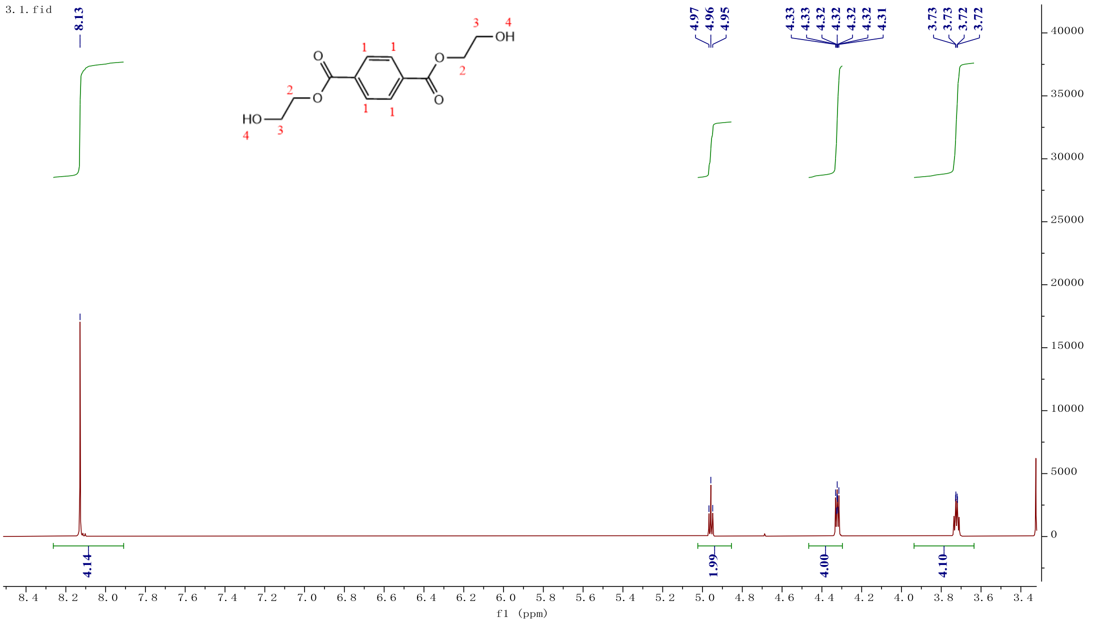

Figure S3 ^{1}H NMR spectrum of BHET

In the HPLC spectrum Fig. S4, only one peak appears other than solvent peak, which indicates that the purity of BHET is high.

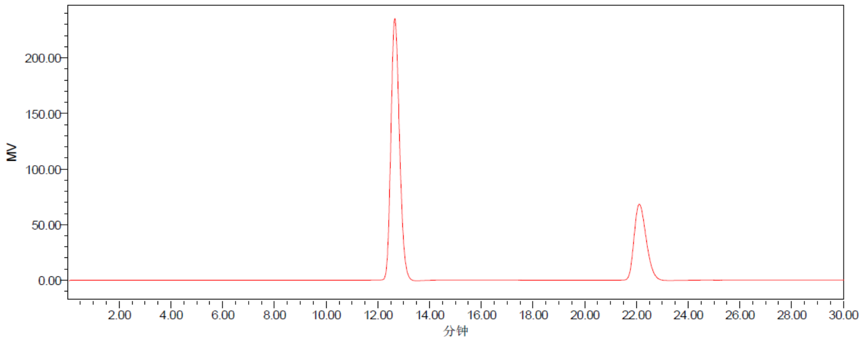

Figure S4 HPLC spectrum of BHET

Fig. S5 demonstrated the TGA curves of the BHET and PET raw material. The PET material experienced only one obvious weight loss process, while BHET experienced two weight loss processes. The TGA curve of PET showed an obvious weight loss at above 390 ℃. The weight loss was more than 80% in the range from 390 ℃ to 461 ℃, which was attributed to the thermal degradation of PET. For BHET, the thermal decomposition appeared in two different steps. The first weight loss at about 250 °C caused by thermal decomposition of BHET. In the second weight loss process, it was found that the weight loss temperature was around 390 ℃, which is consistent with raw PET. It suggested that BHET may be polymerized into PET during the heating process, which is consistent with the literature reports and explains why the second weight loss process was similar to raw PET[6].

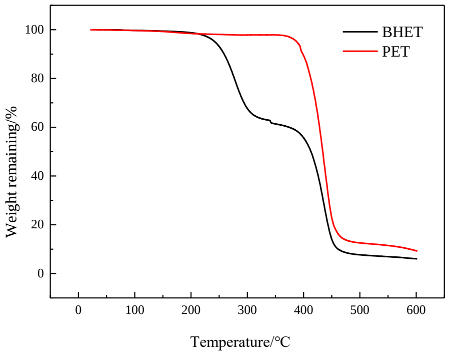

Figure S5 TGA curves of PET raw material and BHET

For BHET, a sharp endothermic peak appears at 110.09 ℃ (Fig. S6). The temperature of this peak is consistent with the melting point of the pure BHET (109.81 °C). The DSC analysis showed that the main product was BHET[7].

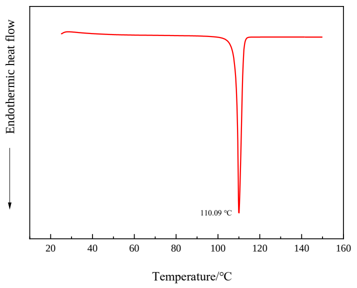

Figure S6 DSC curves of BHET

As shown in Fig. S7, BHET FT-IR spectrum demonstrated strong absorption peaks at 3299.19 cm^{-1} correspond to the characteristic stretching vibration of OH, peaks at 2955.45 and 2873.10 cm^{-1} appeared due to the stretching vibrations of aliphatic $-CH_2-$ group at different position; the strong absorption peaks at 1723.90 and 1271.98 cm^{-1} indicate the presence of ester functional groups, corresponding to the stretching vibrations of C=O and C-O respectively; peaks from 1407 to 1503 cm^{-1} indicate the presence of benzene rings corresponding to the stretching vibration and in-plane bending vibration of C=C in the benzene ring. The transmission bands at 898.23 cm^{-1} and 727.20 cm^{-1} showed p-substitution on the benzene. These peaks are all consistent with reported BHET[8–10].

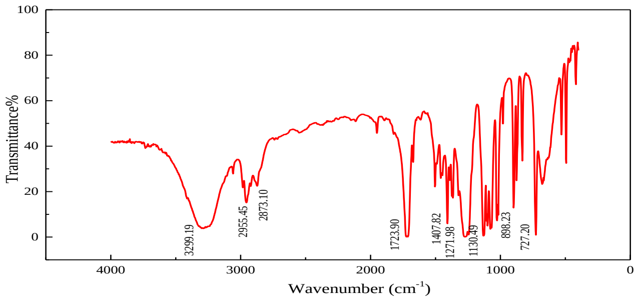

Figure S7 FTIR spectrum of BHET

Section C: Thermal stability analysis of [HDBU][Im]

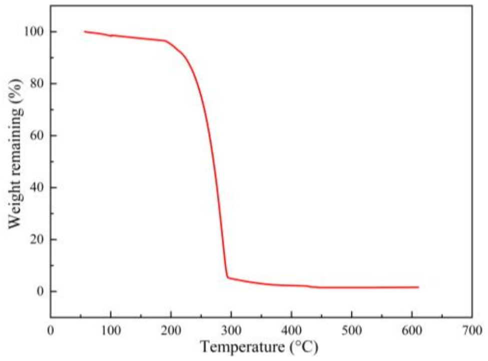

Figure S8 The TGA curve of [HDBU][Im]

Section D: The interaction between DBU and EG

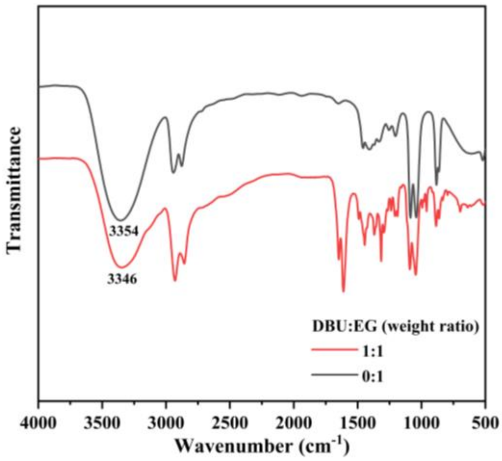

Figure S9 FT-IR spectra of DBU/EG mixtures.

Section E: The crystallinity of PET

FT-IR was used to measure the crystallinity of PET. The absorption peaks observed at 1370 and 1340 cm^{-1} were attributed to the gauche and trans conformations of CH2 wagging in PET, respectively[11]. In amorphous PET, PET mainly exhibits gauche conformation, while in crystalline PET, PET mainly exhibits trans conformation. It is a reliable quantitative method to assess the crystallinity of PET from gauche-to-trans conformation[12,13]. Therefore, the crystallinity of PET could be calculated using the following equation[14,15]:

$$\textit{Trans content ratio} = A_{1340}/(A_{1340}+6.6\ A_{1370}) \quad (1)$$

where A1340 and A1370 are the areas of the absorption bands at 1340 and 1370 cm-1, respectively.

Table S1 The changes in PET crystallinity during the degradation reaction[1]

Reaction time (min)	The crystallinity of PET (%)
0	51.83
10	72.54
20	78.16
30	83.68

[1] Reaction conditions: 5.0 g PET, 5.0 wt% catalyst, 20.0 g EG , at 175°C under 10000 $\mu W\cdot cm^{-2}$ UV radiation.

Reference

[1] Lv Z, Zhang S, Guo Z, et al. Synthesis of alcohol ester 12 in 1, 8-diazabicyclo [5.4.0] undec-7-ene (DBU)-based Self-separation catalytic system. *Applied Organometallic Chemistry* **2019**, *33* (10), e5145, https://doi.org/10.1002/aoc.5145. DOI: https://doi.org/10.1002/aoc.5145 (acccessed 2021/11/02).

[2] Simons W W. *Sadtler handbook of infrared spectra*; Sadtler Research Laboratories, 1978.

[3] Zhang S, Wang J, Liu X, et al. Synthesis of 2, 2, 4-trimethyl-1, 3-pentaerediol monoisobutyrate catalyzed by homogeneous catalysis-liquid/liquid separation catalytic system based on Bibasic sites Ionic Liquids. *Applied Organometallic Chemistry* **2020**, *34* (11), e5915. DOI: https://doi.org/10.1002/aoc.5915.

[4] Liu M, Guo J, Gu Y, et al. Versatile Imidazole-Anion-Derived Ionic Liquids with Unparalleled Activity for Alcoholysis of Polyester Wastes under Mild and Green Conditions. *ACS Sustainable Chemistry & Engineering* **2018**, *6* (11), 15127-15134. DOI: 10.1021/acssuschemeng.8b03591.

[5] Zhu X, Song M,Xu Y. DBU-based protic ionic liquids for CO2 capture. *ACS Sustainable Chemistry & Engineering* **2017**, *5* (9), 8192-8198.

[6] Imran M, Kim D H, Al-Masry W A, et al. Manganese-, cobalt-, and zinc-based mixed-oxide spinels as novel catalysts for the chemical recycling of poly(ethylene terephthalate) via glycolysis. *Polymer Degradation and Stability* **2013**, *98* (4), 904-915. DOI: https://doi.org/10.1016/j.polymdegradstab.2013.01.007.

[7] Wang Q, Geng Y, Lu X, et al. First-Row Transition Metal-Containing Ionic Liquids as Highly Active Catalysts for the Glycolysis of Poly(ethylene terephthalate) (PET). *ACS Sustainable Chemistry & Engineering* **2015**, *3* (2), 340-348. DOI: 10.1021/sc5007522.

[8] Wang Q, Lu X, Zhou X, et al. 1-Allyl-3-methylimidazolium halometallate ionic liquids as efficient catalysts for the glycolysis of poly(ethylene terephthalate). *Journal of Applied Polymer Science* **2013**, *129* (6), 3574-3581. DOI: 10.1002/app.38706.

[9] Al-Sabagh A M, Yehia F Z, Eissa A-M M F, et al. Glycolysis of Poly(ethylene terephthalate) Catalyzed by the Lewis Base Ionic Liquid [Bmim][OAc]. *Industrial & Engineering Chemistry Research* **2014**, *53* (48), 18443-18451. DOI: 10.1021/ie503677w.

[10] Fang P, Liu B, Xu J, et al. High-efficiency glycolysis of poly(ethylene terephthalate) by sandwich-structure polyoxometalate catalyst with two active sites. *Polymer Degradation and Stability* **2018**, *156*, 22-31. DOI: https://doi.org/10.1016/j.polymdegradstab.2018.07.004.

[11] Matsuo, M, Luo, Y, Galeski, A. Gauche-trans transitions in amorphous polymers under annealing: Lattice model and polarized light scattering. *Physical Review E* **2009,** *79* (4), 041801.

[12] Zhang, Y, Lu, Y, Duan, Y, Zhang, J, Yan, S, Shen, D. Reflection–absorption infrared spectroscopy investigation of the crystallization kinetics of poly (ethylene terephthalate) ultrathin films. *Journal of Polymer Science Part B: Polymer Physics* **2004,** *42* (24), 4440-4447.

[13] Cole, K C, Ajji A, Pellerin E. New insights into the development of ordered structure in poly (ethylene terephthalate). 1. Results from external reflection infrared spectroscopy. *Macromolecules* **2002,** *35* (3), 770-784.

[14] Wang Y, Shen D, Qian R. Subglass-transition-temperature annealing of poly (ethylene terephthalate) studied by FTIR. *Journal of Polymer Science Part B: Polymer Physics* **1998,** *36* (5), 783-788.

[15] Qian R, Shen D, Sun F, Wu L. The effects of physical ageing on conformational changes of poly (ethylene terephthalate) in the glass transition region. *Macromolecular Chemistry and Physics* **1996,** *197* (4), 1485-1493.
